# Phenotype microarrays reveal metabolic dysregulations of neurospheres derived from embryonic Ts1Cje mouse model of Down syndrome

**DOI:** 10.1371/journal.pone.0236826

**Published:** 2020-07-30

**Authors:** Eryse Amira Seth, Han-Chung Lee, Hadri Hadi bin Md Yusof, Norshariza Nordin, Yoke Kqueen Cheah, Eric Tatt Wei Ho, King-Hwa Ling, Pike-See Cheah

**Affiliations:** 1 Genetics & Regenerative Medicine Research Centre (GRMRC), Faculty of Medicine and Health Sciences, Universiti Putra Malaysia, Serdang, Selangor, Malaysia; 2 Department of Human Anatomy, Faculty of Medicine and Health Sciences, Universiti Putra Malaysia, Serdang, Selangor, Malaysia; 3 Department of Biomedical Science, Faculty of Medicine and Health Sciences, Universiti Putra Malaysia, Serdang, Selangor, Malaysia; 4 Center for Intelligent Signal & Imaging Research, Universiti Teknologi Petronas, Bandar Seri Iskandar, Perak, Malaysia; IGBMC/ICS, FRANCE

## Abstract

Down syndrome (DS), is the most common cause of intellectual disability, and is characterized by defective neurogenesis during perinatal development. To identify metabolic aberrations in early neurogenesis, we profiled neurospheres derived from the embryonic brain of Ts1Cje, a mouse model of Down syndrome. High-throughput phenotypic microarray revealed a significant decrease in utilisation of 17 out of 367 substrates and significantly higher utilisation of 6 substrates in the Ts1Cje neurospheres compared to controls. Specifically, Ts1Cje neurospheres were less efficient in the utilisation of glucose-6-phosphate suggesting a dysregulation in the energy-producing pathway. T Cje neurospheres were significantly smaller in diameter than the controls. Subsequent preliminary study on supplementation with 6-phosphogluconic acid, an intermediate of glucose-6-phosphate metabolism, was able to rescue the Ts1Cje neurosphere size. This study confirmed the perturbed pentose phosphate pathway, contributing to defects observed in Ts1Cje neurospheres. We show for the first time that this comprehensive energetic assay platform facilitates the metabolic characterisation of Ts1Cje cells and confirmed their distinguishable metabolic profiles compared to the controls.

## Introduction

Down syndrome (DS), or Trisomy 21, is a genetic disorder that results from the partial or full triplication of human chromosome 21 (HSA21), which leads to multiple phenotypes with varying complexity. The frequency of DS worldwide is approximately 1 in 1000 live births [1], whereas, in Malaysia, the prevalence of DS is approximately 1 in 660 live births (Kiwanis Down Syndrome Foundation, KDSF, https://www.kdsf.org.my/what-is-down-syndrome/). Clinical manifestations that are prominent in individuals with DS include intellectual disability, craniofacial abnormalities and muscle weakness.

In recent years, the development of various mouse models for DS has been indispensable in enhancing our knowledge of the molecular mechanisms and ensuing complex phenotypes observed in DS. The partial homology between HSA21 and mouse chromosome 16 (*Mus musculus* 16, MMU16), chromosome 10 (MMU10) and chromosome 17 (MMU17) have been an impetus for the generation of mouse models with DS [2]. Developed in 1998 by Sago and colleagues, the Ts1Cje mouse model carries ~80 genes of MMU16 that are homologous to HSA21. This partially trisomic mouse model was reported to display DS-associated behavioural deficits and neuropathologies including hippocampus-related learning and memory impairment [3], muscle weakness [4] as well as reduced cerebellar volume [5,6].

Neural stem and progenitor cells (NSPCs) is the collective term for a population of stem cells that can self-renew and also progenitor cells that are more committed to either the neuronal or glial lineage [7]. The process of generation from NSPCs to neurones and glial cells are known as neurogenesis and gliogenesis, respectively. Both processes can occur during prenatal, postnatal and adult stages in the normal mouse brain [7–10] A study using an *in vitro* method, known as the neurosphere culture, which derived from neural stem cells (NSCs) have contributed to the existing knowledge on proliferative capacity and cell fate determination [11]. Hewitt and colleagues reported a reduced proportion of neurones and an increased proportion of astrocytes derived from adult Ts1Cje neurospheres [12]. Dysregulated JAK-STAT signaling pathway was then implicated in this neurogenic-to-gliogenic shift in embryonic Ts1Cje mice [13,14]. A higher proportion of cells positive for the immunohistochemical marker for glial cells, glial fibrillary acidic protein (GFAP) was observed when neurospheres from the embryonic neocortex of Ts1Cje mice were allowed to differentiate ^15^. The same study reported a decreased rate of proliferation in embryonic NSCs derived from Ts1Cje mice compared to control. Taken together, it is indisputable that neurogenesis defects occur in the brain of mouse models of DS at very early stages of development and understanding the underlying mechanisms responsible for that would be useful for future interventional therapies.

Metabolic studies of DS brain have shown that dysregulated level of metabolites and impaired glucose metabolism in the brain of individuals with DS are correlated with the progression of cognitive dysfunction in DS [15–18]. Recently, perturbed metabolic profiles associated with muscle weakness are reported in the adult Ts1Cje mouse model of Down syndrome [19]. However, studies on the metabolic properties of embryonic NSCs in mouse models, particularly Ts1Cje mice, are still limited.

Biolog Phenotype MicroArray (PM) is a technology that provides a cellular analysis of multiple physiological traits simultaneously, using a 96-well microplate pre-coated with different known substrates such as carbon sources, L-amino acids and dipeptides [20]. This strategy incorporated the redox technology with cells respiration (NADH production) as a universal reporter. When the seeded cells respire actively, this aerobic metabolism will reduce the tetrazolium dye yielding a strong purple-coloured formazan dye at 37°C. The formazan production can subsequently be quantified by an endpoint absorbance at 590 nm with a temperature-controlled Omnilog Microplate Reader [21].

In this study, we used the PM technology to pinpoint the metabolic capacity of Ts1Cje neurospheres towards energy metabolism substrates that led to the identification of potentially altered metabolic pathways underlying the defective proliferation and neurogenesis observed in embryonic Ts1Cje mice. We then further validated the dysregulations in energy-producing metabolic pathways of Ts1Cje neurospheres via a supplementation assay.

## Materials and methods

### Animal husbandry

The Ts1Cje breeders were revived from cryosperm bank of Walter and Eliza Hall Institute of Medical Research (WEHI), Australia. Ts1Cje males of C57BL/6 background were mated with wild type (WT) C57BL/6 females. The Ts1Cje mice used in this study were bred between 7^th^ and 9^th^ generations from the original colony in WEHI. All mice were housed in the mouse room facility of Genetics and Regenerative Medicine Research Centre (GRMRC), Faculty of Medicine and Health Sciences, Universiti Putra Malaysia, under controlled temperature (21–23°C) with a 12:12 hour light-dark cycle. The mice were given unlimited access to standard animal feed (Altromin 1324, Germany) and clean water *ad libitum*. All experiments that involved animal breeding and handling were approved by the Institutional Animal Care and Use Committee of Universiti Putra Malaysia and were performed in accordance to the institutional regulations on experimental animals (Reference number: UPM/IACUC/AUP-R003/2014).

### Mouse genotyping analysis

Genomic DNA was extracted from the tail samples and was genotyped as described [22]. In brief, gDNA sample with the A260/280 ratio between 1.7–1.9 was subjected to PCR using two sets of primers; the gl0utamine receptor ionotropic kainite 1 (*Grik1*)(Forward, 5’-CCCCTTAGCATAACGACCAG-3’; Reverse, 5’- GGCACGAGACAGACACTGAG-3’) and neomycin (*Neo*) (Forward, 5’-CTCACCTTGCTCCTGCCGAG-3’; Reverse, 5’- CTGATGCTCTTCGTCCAGATCATC-3’) were used to perform PCR for genotyping analysis as described previously [3]. One of the three copies of *Sod1* gene was disrupted by the neomycin resistance sequence located at the trisomic segment of MMU16, therefore the *Neo3* and *Neo4* primers were used, along with *Grik1-F* and *Grik1-R* as the internal controls.

### Neurospheres culture

For timed breeding, two WT C57BL/6 females were housed with a Ts1Cje male for overnight. The female mice were observed for the presence of a vaginal plug the following morning. The day a vaginal plug was observed was considered as embryonic day 0.5 (E0.5). Developmental stages of embryos were further determined based on the Theiler Stage Definition from The e-Mouse Atlas (http://www.emouseatlas.org/emap/ema/theiler_stages/theiler_stages.html). At E15.5, both the fingers and toes are separated and clearly divergent, but not becoming parallel until a later stage of development. Cerebral cortical tissues were microdissected from the embryonic (E) day 15.5 pups and were mechanically dissociated to form a single cell suspension. The supernatant was removed and the cell pellet was resuspended in the complete neurosphere culture medium [Neurobasal™ Medium (Gibco, USA), supplemented with 1X of B-27® Supplement (Gibco, USA), 1% of GlutaMAX™ Supplement, 1% of Penicillin/Streptomycin (Gibco, USA), 20 ng/ml of epidermal growth factor (EGF, Thermo Fisher Scientific) and 20 ng/ml of basic fibroblast growth factor (bFGF, Thermo Fisher Scientific)]. The cells were then cultured in complete neurosphere culture medium in T25 flask at a density of 5x10^5^ cells/ml and incubated at 37°C in a humidified incubator with 5% CO_2_. After 5 to 7 days *in vitro* (DIV), the proliferating NSPCs generate non-adherent spherical clusters of cells, termed neurospheres, which measure 100 to 200 μm in diameter. At this stage, the neurospheres can either be passaged or subjected to downstream metabolic profiling experiments.

### Biolog Phenotype MicroArray (PM)

A total of twelve different E15.5 embryos per genotype were obtained from five separate litters, to generate neurospheres for Phenotype MicroAssay experiments. For each PM-M plate, the neurospheres generated from wild type (n = 3) and Ts1Cje (n = 3) embryos were derived from three different litters. Neurospheres were trypsinised using 0.05% trypsin-EDTA (Gibco, USA) and were mechanically dissociated into a single-cell suspension. The cells were pelleted, resuspended in MC-0 assay media [consists of 1X Biolog IF-M1 media (Biolog, USA), 0.3mM L-Glutamine (Gibco, USA) and 1% Penicillin/Streptomycin (Gibco, USA)]. The Biolog PM assay was performed in biological triplicates. Metabolic profiling of embryonic neurospheres were performed using four mammalian Phenotype MicroArray (PM-M) microplates; PM-M1, PM-M2, PM-M3 and PM-M4. These 96-well microplates are pre-coated with a total of 367 oxidizable carbon sources. PM-M1 is pre-coated with various simple reducing sugars, carbohydrates and carboxylates, whereas PM-M2, PM-M3 and PM-M4 wells are pre-coated with L-amino acids and various dipeptides [20]. For each well, 2x10^4^ cells were loaded and mixed with Redox Dye Mix MA (Biolog, USA) followed by incubation in Biolog’s Omnilog PM instrument at 37°C. The OmniLog reader was set to measure the tetrazolium reduction, at 15-minute intervals for 48 hours. The data collected by the OmniLog® Phenotype MicroArray (PM) system was organised using the OmniLog^®^ PM software (Biolog, USA). Subsequently, the File Management/Kinetic Analysis program was used to assemble the data for each microplate into an integrated dataset and was subjected to a downstream data analysis pipeline.

### Data analysis pipeline using R

Data analysis for Biolog PM results was executed in the R statistical software (www.r-project.org, version R-3.2.0) based on the R script (http://www.helsinki.fi/bsg/software/R-Biolog/) as previously described [23]. In brief, the analysis pipeline involves three steps: grouping, normalisation and effect identification. The metabolic signals for each well were first corrected against the background signal produced by the negative control well, which contains no substrate. In the grouping step, the metabolic profiles were separated into “active metabolic profiles” (wells that show positive metabolic signals, indicating substrate is utilised by the cells and there is sufficient energy supply enabling cell respiration) and; “non-active metabolic profiles” (wells that show no metabolic signal, represents the lack of the ability to catabolise that substrate). A threshold value of 20 OmniLog unit was selected for initial grouping into the active and non-active metabolic profiles, in which this value was high enough to ensure that the positive control value remains positive and also low enough of a value to ensure that the negative control value remains negative. Profiles that exceed the threshold were labelled as active group and the rest were assigned to the non-active group. The active profiles were fitted to a logistic model (y_t_ = asym/1+exp (xmid-x)/scal, *t = time point at which a metabolic signal is measured; asym = metabolic signal as t→* ∞; *xmid = time at which Asym/2; scale = inverse of the maximum growth rate*), whereas the non-active profiles were fitted to a linear model (y_t_ = b_0_ + b_1_*t; *t = time point at which a metabolic signal is measured; b*_*0*_ *= starting level; b*_*1*_ *= slope*).

After initial grouping, the Expectation-Maximization algorithm was applied, which iteratively classified the profiles by fitting them into the logistic and linear models. If it failed to fit the active profile to a logistic model, as previously fitted from the initial threshold grouping, then a linear model was fitted. Subsequently, the normalisation step involves making the biological triplicates comparable with each other. Normalisation was performed by generating a base active curve for active and non-active profiles separately. Similar to the grouping step, the base active curve for the active profiles was generated by fitting to a logistic curve and the base active curve for non-active profiles was fitted to a linear curve. Following generation of base active curves, the metabolic profiles of one replicate remain unchanged to serve as a reference, and the metabolic profiles of the other two replicates were normalised against the reference.

For the effect identification step, the individual substrate profile was first fitted to either a logistic or linear models similar to grouping and normalisation steps. The fitted models in this step describe each substrate, as opposed to describing a group of profiles in grouping and normalisation steps. The fitted value for each time point was used as data for the Bayesian hierarchical variance analysis. This statistical model analyses the statistical significance of the comparison between metabolic signals of Ts1Cje and WT groups. The significance is expressed as positive, where Ts1Cje metabolic signal is lower than WT. On the other hand, the significance is expressed as negative, where Ts1Cje metabolic signal is higher than WT and is considered insignificant when no significant differences in metabolic signals between the two groups. The threshold value was then used to separate the active and non-active metabolic profiles. Multiple different threshold values were applied to the analysis pipeline across all PM-M plates until the optimal number of differentially utilised substrates was achieved.

### 6PGA supplementation assay, phenotypic readout of the neurospheres and statistical analysis

Ten milligrams of 6-phosphogluconic acid trisodium salt (6PGA) (Sigma-Aldrich, USA) were reconstituted in autoclaved distilled water to achieve a stock solution with a final concentration of 50 mM. Neurospheres measuring 100 to 200 μm were dissociated into a single cell suspension and were seeded at a density of 2.5 x 10^4^ cells per well. The cells were supplemented with 1.0 mM and 2.0 mM of 6PGA and incubated for 6 days at 37°C in a humidified incubator with 5% CO_2_. Under the brightfield microscope, each well was equally divided into four quadrants. One micrograph from each quadrant was randomly taken and the total number of neurospheres in all four micrographs was calculated. Subsequently, ImageJ software (https://imagej.nih.gov/ij/index.html) was used to measure the diameter of each neurosphere from all four micrographs. This quantitative analysis was repeated for all three biological replicates for each group, Ts1Cje and WT neurospheres. Data shown represent mean ± S.E.M. Two-way ANOVA followed by Sidak’s multiple comparisons correction analysis of the neurosphere diameter mean values for each biological replicate were performed using Prism 7 (Graphpad Software Inc., USA). Corrected *p*-values <0.05 were considered statistically significant.

## Results

### PM analysis

**Fig 1:**
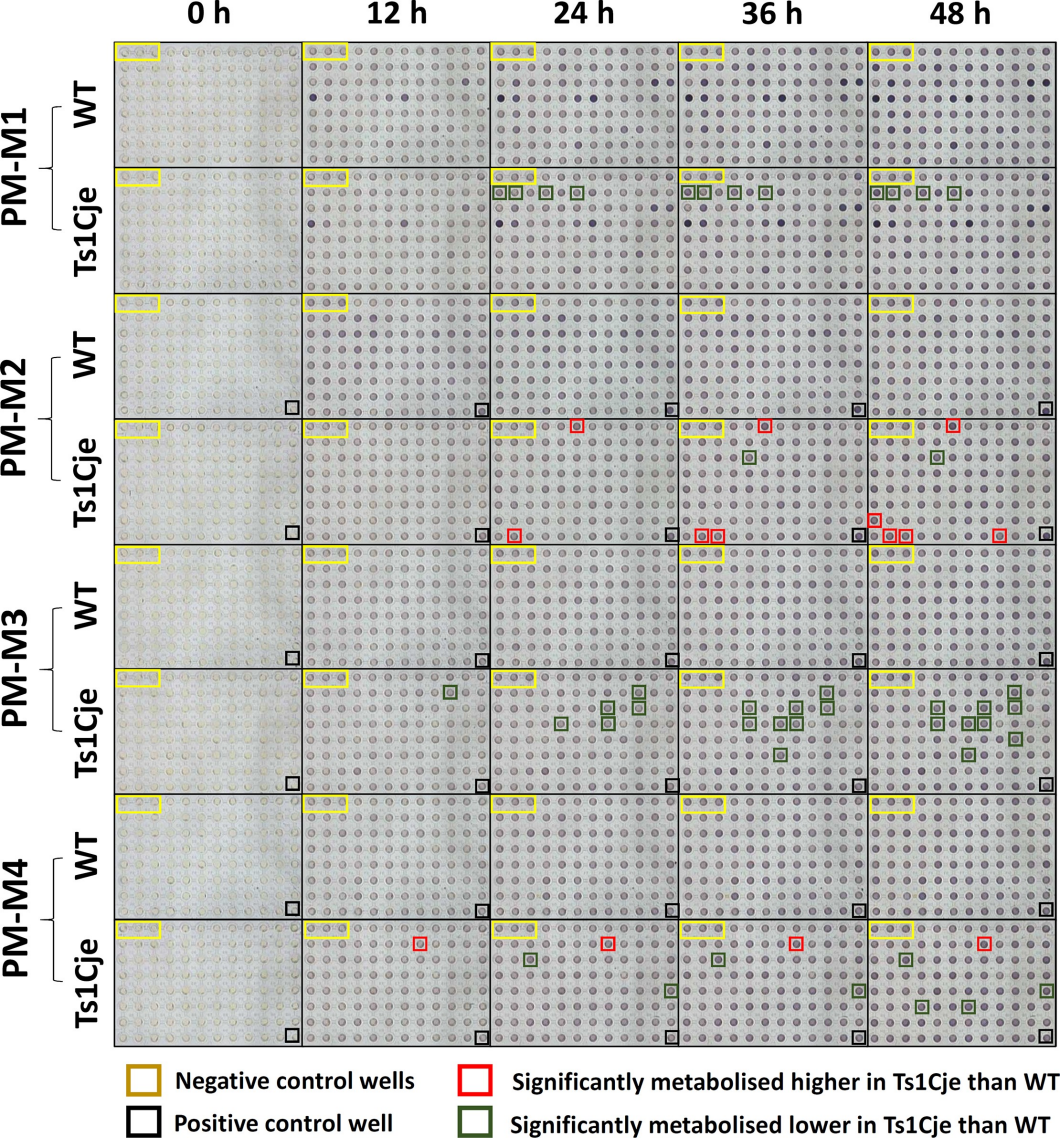
Distinctive patterns of substrate utilization between Ts1Cje and WT neurospheres. Using plates PM-M1 through PM-M4, distinctive patterns of 48-hour substrate utilisation were observed between Ts1Cje and WT neurospheres, as indicated in red- and green-coloured boxes. The yellow boxes (well positions A1 to A3, in PM-M1 to PM-M4) represent the negative control wells, the black box (well positions H12, in PM-M2 to PM-M4) represents positive control well.

**Fig 2:**
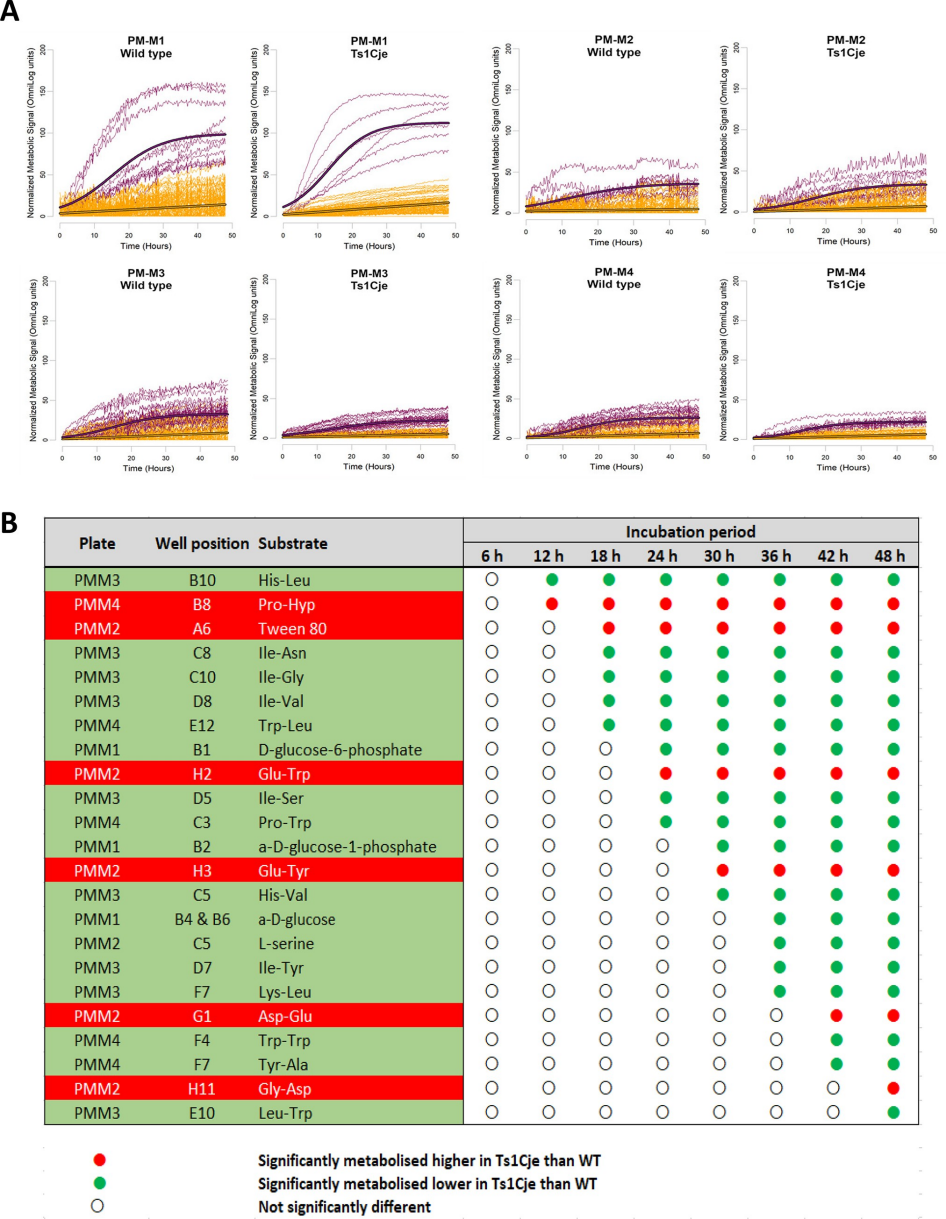
Normalised metabolic profiles of WT and Ts1Cje-derived neurospheres for plate PM-M1 to PM-M4. **(A)** Normalised metabolic profiles of WT and Ts1Cje-derived neurospheres for plate PM-M1 to PM-M4. Each curve represents the metabolic signal produced in each well. The purple lines correspond to active profiles, whereas the yellow lines represent non-active profiles. The thick purple line represents the mean asymptote of active base curve. The x-axis and y-axis represent time in hours and normalised metabolic signal, respectively. **(B)** The pattern of the significantly utilised substrates changes with time captured at 6-hour intervals over the 48-hour incubation period.

The ability of WT and Ts1Cje neurospheres to utilise basic elemental nutrients such as carbon, nitrogen, phosphorus and sulfur-based substrates was determined using four types of PM-M microplates pre-coated with a total of 367 substrate nutrients, as indicated by colour changes throughout the 48-hour incubation period (Fig 1). Representative plots of active and non-active metabolic profiles for plates PM-M1 to PM-M4 are shown in Fig 2A. For plate PM-M1, the mean asymptote of Ts1Cje active base curve was higher than the mean asymptote of WT active base curve. In contrast, the mean asymptote of Ts1Cje active base curves in plates PM-M2 and PM-M3 were lower than that of the WT active base curves. Meanwhile, the mean asymptote of Ts1Cje active base curve in plate PM-M4 was slightly higher than that of WT active base curve. On average, the number of active profiles in plate PM-M1 for Ts1Cje neurospheres is 10, which is similar to the number of active profiles of WT neurospheres. The average number of active profiles in plate PM-M2 for Ts1Cje was higher compared to WT control. On the other hand, a lower number of active profiles were observed for Ts1Cje neurospheres in plates PM-M3 and PM-M4 as compared to WT neurospheres. The values of base curve parameters and the number of active and non-active metabolic profiles are shown in Table 1. Raw data for PM-M1 through PM-M4, for both Ts1Cje and WT neurospheres, on the grouping of the metabolic profiles and their respective fitted models (either linear or logistic) was tabulated in S1 File.

**Table 1:** Summary of metabolic profiles of wildtype (WT) and Ts1Cje neurospheres for Biolog Phenotypic plates, PM-M1 to -M4.

	PM-M1		PM-M2		PM-M3		PM-M4	
	WT	Ts1Cje	WT	Ts1Cje	WT	Ts1Cje	WT	Ts1Cje
**Number of active metabolic profiles**	10 ± 1	10 ± 3	12 ± 1	18 ± 6	40 ± 8	31 ± 5	27 ± 6	22 ± 11
**Number of non-active metabolic profiles**	83 ± 1	83 ± 3	81 ± 1	75 ± 6	53 ± 8	62 ± 5	66 ± 6	71 ± 11
**Asymptote of active base curve (Omnilog units)**	100.03 ± 6.88	117.79 ± 15.25	35.92 ± 4.74	31.09 ± 5.85	34.47 ± 8.21	25.32 ± 6.53	22.51 ± 3.07	23.30 ± 10.54
**Slope of non-active base curve**	0.26 ± 0.07	0.38 ± 0.21	0.07 ± 0.04	0.16 ± 0.09	0.26 ± 0.17	0.13 ± 0.09	0.13 ± 0.04	0.24 ± 0.17

Data shown represent the mean of replicates (n = 3 per group) ± S.E.M.

### Significant differentially utilised substrates

The effect identification step of the data analysis pipeline was performed to identify substrates that were significantly differentially utilised by Ts1Cje neurospheres as compared to that of WT. Values fitted to either logistic or linear curves (obtained from the grouping and normalisation steps) at 8 different time points (6, 12, 18, 24, 30, 36, 42 and 48 hours) serve as data for the Bayesian hierarchical variance analysis. Based on the threshold value of 20, the statistical significance of the differentially utilised substrates was observed for a total of 23 substrates over the period up to 48 hours of incubation (Fig 2B). Raw absorbance data for PM-M1 through PM-M4 for both Ts1Cje and WT neurospheres are provided (S1 to S4 Raw data).

In PM-M1, which contains primarily carbohydrate and carboxylate substrates, the metabolism of D-glucose-6-phosphate, α-D-glucose-1-phosphate and α-D-glucose were significantly poorly utilised by Ts1Cje neurospheres compared to WT neurospheres between 24 and 30 hours. PM-M2 to PM-M4, on the other hand, contain lipids and protein-derived nutrients, primarily amino acids and dipeptides. In PM-M2, a total of 6 substrates were significantly differentially utilised by neurospheres of both genotypes. Ts1Cje neurospheres showed lower metabolism of L-serine than that of WT neurospheres. In contrast, Ts1Cje neurospheres demonstrated stronger metabolism of Tween 80, aspartyl-glutamate (Asp-Glu), glutamyl-tryptophan (Glu-Trp), glutamyl-tyrosine (Glu-Tyr) and glycyl-aspartic acid (Gly-Asp) when compared to that of WT neurospheres. The highest number of significant differentially utilised substrates was detected in PM-M3, with 9 dipeptides that were less metabolised by Ts1Cje neurospheres as compared to WT. These substrates include histidyl-leucine (His-Leu), histidyl-valine (His-Val), isoleucyl-asparagine (Ile-Asn), isoleucyl-glycine (Ile-Gly), isoleucyl-serine (Ile-Ser), isoleucyl-tyrosine (Ile-Tyr), isoleucyl-valine (Ile-Val), leucyl-tryptophan (Leu-Trp), lysyl-leucine (Lys-Leu). For PM-M4, 4 dipeptides, namely prolyl-tryptophan (Pro-Trp), tryptophyl-tryptophan (Trp-Trp), tyrosyl-alanine (Tyr-Ala) and tryptophyl-leucine (Trp-Leu) were significantly poorly metabolised by Ts1Cje neurospheres as compared to WT neurospheres. On the other hand, Ts1Cje neurospheres demonstrated significantly stronger metabolism in the well containing prolyl-hydroxyproline (Pro-Hyp) as compared to WT neurospheres (Fig 2B).

Fig 2B indicates the significant differentially utilised substrates at 8 different time points: 6, 12, 18, 24, 30, 36, 42 and 48 hours. It is an interesting notion that some substrates were significantly utilised shortly after 12 hours of incubation whereas some required extended incubation time (up to 48 hour). As early as 12 hours of incubation, two dipeptides, namely His-Leu and Pro-Hyp were significantly utilised at all time points thereafter. After 18 hours of incubation, Tween 80 and four dipeptides (Ile-Asn, Ile-Gly, Ile-Val and Trp-Leu) were significantly utilised until 48 hours of incubation. As much as 71% of the significant differentially utilised substrates showed significance at 24 hours and above. Of that, neurospheres acquired rather long incubation time (between 42 to 48 hours) to exhibit significant utilisation on two dipeptides, namely Gly-Asp and Leu-Trp (Fig 2B).

### Generation of neurospheres and preliminary study on 6PGA supplementation assay

**Fig 3:**
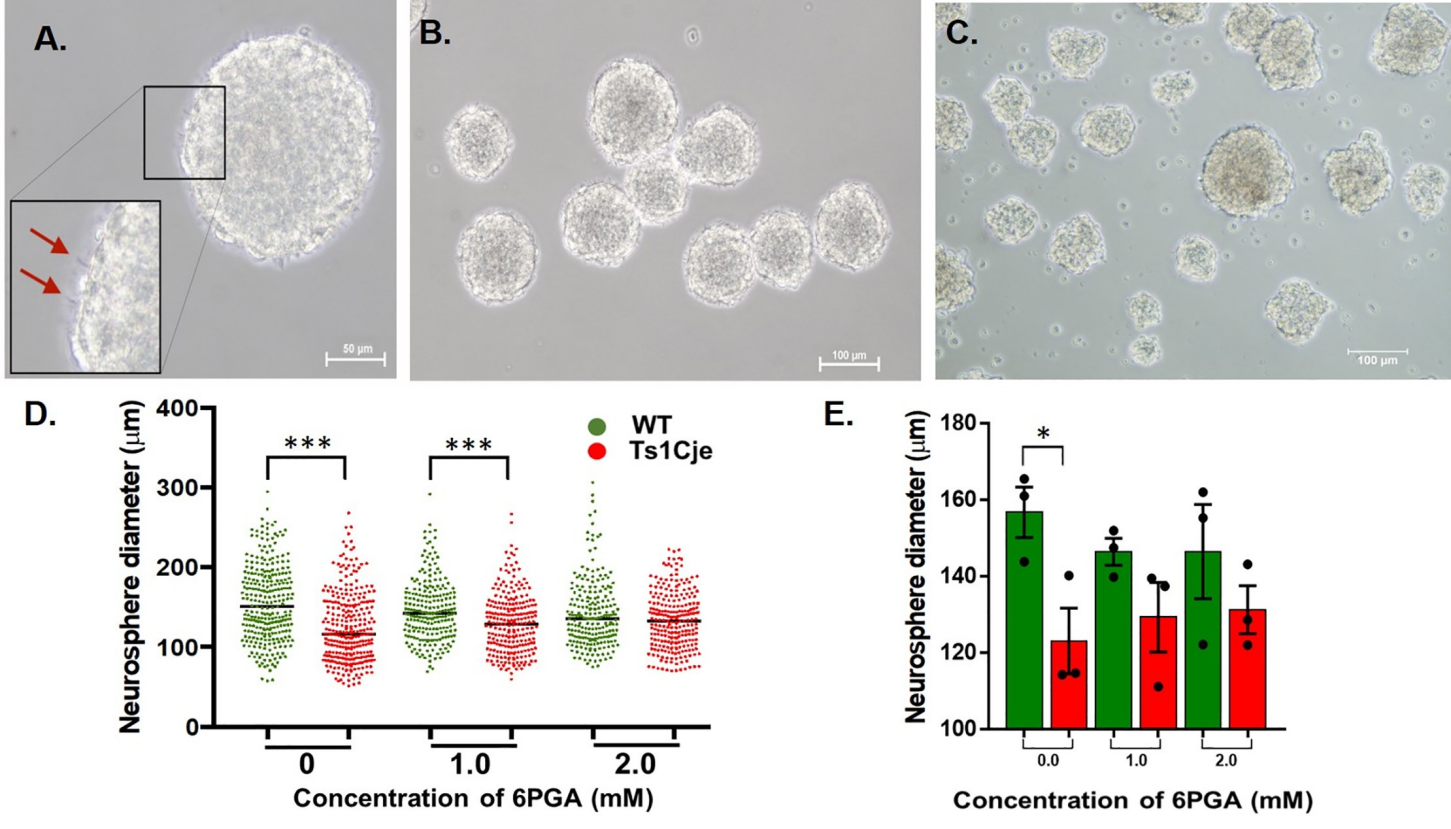
Effect of 6PGA supplementation on neurosphere diameter. **(A)** A healthy neurosphere is spherical in shape, with microspikes (red arrows) observed on the outer surface: magnification, X200. Neurospheres derived from cerebral cortices of WT **(B)** and Ts1Cje embryos **(C)**, cultured at day 5 *in vitro*. Magnification, X100. The size of neurospheres derived from three biological replicates supplemented with 6PGA is represented in a scatter plot **(D)** (n = 288 vs 296, 227 vs 240 and 224 vs 249 for WT vs Ts1Cje for 0 mM, 1.0 mM and 2.0 mM 6PGA supplementations, respectively) whereas the mean measurement for each biological replicates is represented in a bar chart **(E)** (n = 3 per group per treatment). Each dot represents a neurosphere (D) or a biological replicate (E). Two-way ANOVA with Sidak’s multiple comparisons correction was performed on both analyses in (D) and (E). Black bars represent the mean diameter for each group. * denotes adjusted *p*<0.05 whereas *** denotes adjusted *p*<0.001.

Neurospheres are aggregates composed of a heterogeneous population of NSPCs. NSPCs isolated from the WT E15.5 embryonic cerebral cortices, when cultured in media supplemented with EGF and FGF, generated healthy and viable neurospheres exhibited with microspikes on the outer surface (Fig 3A), with a diameter measured between 100 μm to 200 μm (Fig 3B). Ts1Cje neurospheres were significantly (P < 0.05) smaller in size compared to that of the WT (Fig 3C and 3D). Based on the Biolog PM experiments, Ts1Cje neurospheres have significantly lower metabolism of G6P compared to WT neurospheres. We then performed a preliminary study to further validate the G6P metabolism in Ts1Cje neurospheres and, they were supplemented with 6PGA, the intermediates of G6P metabolism in the pentose phosphate pathway (PPP). Neurospheres were supplemented with 0 mM, 1.0 mM and 2.0 mM of 6PGA for 6 days *in vitro*, and their size was measured (Fig 3D). Without 6PGA supplementation, the mean diameter of Ts1Cje neurospheres was 124.2 ± 2.6 μm (n = 296), whereas the mean diameter of WT neurospheres was 155.40 ± 2.8 μm (n = 288). Ts1Cje neurospheres, without 6PGA supplementation, was 21% significantly smaller than the WT neurospheres (P <0.0001; Fig 3D. When supplemented with 1.0 mM of 6PGA, the mean diameters of Ts1Cje and WT neurospheres were 130.0 ± 2.4 μm (n = 240) and 146.4 ± 2.5 μm (n = 227), respectively (Fig 3D). There was a 12% reduction in the mean diameter of Ts1Cje neurospheres compared to WT neurospheres with the difference was statistically significant (P < 0.0001) (Fig 3D). When treated with 2.0 mM of 6PGA, the mean diameter of the neurospheres measured 133.3 ± 2.2 μm (n = 249) and 141.5 ± 2.8 μm (n = 224) for Ts1Cje and WT neurospheres, respectively. Ts1Cje neurospheres were 10% smaller than WT neurospheres when supplemented with 2.0 mM and the difference was not statistically significant (P = 0.0907) (Fig 3D). The mean measurement of neurosphere for each biological replicates is represented in a bar chart (Fig 3E). There was a significant difference between the untreated WT and Ts1Cje groups (P = 0.0393) but not in 6PGA supplemented groups In summary, increased supplementation with 6PGA demonstrated a trend of rescuing the phenotypic readout of the Ts1Cje neurospheres, achieving similar mean diameter as seen in the WT neurospheres.

## Discussion

The present study has revealed significant differences in the total number of active and non-active profiles in Ts1Cje neurospheres as compared to WT neurospheres suggesting that WT and Ts1Cje neurospheres exhibit significantly different patterns of substrate utilisation. To the best of our knowledge, this study is the first to provide data on metabolic profiling of Ts1Cje neurospheres using Biolog Phenotype MicroArray and also data analysis using the R pipeline established by Vehkala and colleagues [23]. The active substrates that were metabolised by both WT and Ts1Cje neurospheres include different combinations of monosaccharides, disaccharides, ketoses, carboxylic acids, L-amino acids and dipeptides. Animal cells can metabolise substrates other than glucose for energy production [20]. The present results, therefore, suggest that NSPCs derived from both embryonic WT and Ts1Cje can utilise substrates other than glucose as alternative energy sources, and Ts1Cje more than WT. The data from the analysis pipeline revealed a total of 23 significantly differentially utilised substrates, with 17 significantly less and 6 more in Ts1Cje neurospheres compared to WT neurospheres.

Glucose-1-phosphate (G1P) is an intermediate in the breakdown of glycogen (glycogenolysis) and is converted to glucose-6-phosphate (G6P) by the enzyme phosphoglucomutase [24]. G6P can subsequently be catabolised along with the glycolysis or PPP. Although glycogen metabolism has been implicated in the maturation of astrocytes, little is known about the specific role of G1P in NSPCs [25]. Since G1P can be converted to G6P, there is a possibility that the neurospheres were able to utilise G1P as an alternative energy source. The present data showed that G1P metabolism is significantly lower in the Ts1Cje neurospheres, suggesting that they possibly utilised G1P as an alternative source less effectively compared to the WT neurospheres.

Other significant differentially utilised substrates include those that are involved in glucose metabolism, specifically glycolysis and PPP. Metabolism of α-D-glucose and D-glucose-6-phosphate were significantly lower in Ts1Cje neurospheres compared to WT neurospheres, with a significantly larger difference in metabolism at later time points. Glucose is the main energy-producing substrate in the brain. Glucose metabolism provides precursors for the synthesis of neurotransmitters and ATP for the brain’s metabolic demands [26]. Low exposure to glucose resulted in decreased proliferation of NSCs derived from E15.5 mouse brain, but did not have an effect when the NSCs were subsequently allowed to differentiate with 1% FBS [27]. This study suggests that glucose utilisation is vital in self-renewal and maintenance of progenitor pool but less critical for differentiation into mature cells. Although evidence on glucose metabolism in Ts1Cje mice is currently limited, the lower glucose metabolism in the Ts1Cje neurospheres observed in this present study is consistent with the decreased cerebral glucose metabolism found in DS patients with dementia prior to the onset of any symptoms, and the metabolic rate declined with the severity of AD [17]. The mechanism by which the glucose metabolism is dysregulated in Ts1Cje is, however, remains unclear. The metabolic profiling of differentiating NSPCs derived from embryonic Ts1Cje neurospheres warrants further investigation.

Another prominent finding in this current study is the significantly reduced metabolism of L-serine in the Ts1Cje neurospheres. L-serine is a non-essential amino acid that exhibits essential functions in the central nervous system, particularly protein synthesis and cell proliferation [28]. For example, treatment with L-serine facilitated the proliferation of NSCs derived from rats with induced brain injury, and pharmacological inhibition of the enzyme serine hydroxymethyltransferase (SHMT) impaired the effects of L-serine on NSC proliferation [29]. SHMT initiates L-serine catabolism in the one-carbon metabolism for purine and pyrimidine synthesis [28], suggesting that L-serine possibly acts on NSPCs through this pathway. Work by Stříšovský and colleagues [30] also found an alternative catabolic pathway of L-serine, where the enzyme serine racemase in the mouse brain eliminates water from L-serine to form pyruvate. Pyruvate can then be fed into the Krebs cycle for ATP production [31]. To date, there is lack of evidence for the specific role of L-serine in NSPCs derived from embryonic Ts1Cje mice. Previous studies have demonstrated decreased levels of serine in the plasma of patients with DS, suggesting the involvement of dysregulated serine metabolism in DS neuropathology [32,33]. However, it is unclear whether lower level of serine directly indicates impaired serine metabolism in NSPCs. Since the Biolog PM technology measures the NADH produced from substrate utilisation, it is hypothesised that L-serine is metabolised by serine racemase for energy production and that this energy-producing pathway is impaired in Ts1Cje neurospheres. Further experiments on the conversion of L-serine to pyruvate in NSPCs derived from Ts1Cje would be required to confirm this postulation.

The present study has implicated that five isoleucine-containing dipeptides (Ile-Asn, Ile-Gly, Ile-Ser, Ile-Tyr and Ile-Val) were utilised significantly lower in the Ts1Cje neurospheres compared to the WT neurospheres. Lower level of isoleucine was previously reported in the plasma of adults with DS compared to healthy controls [33]. Isoleucine, an essential amino acid, is a branched chain amino acid (BCAA) that has essential functions in protein synthesis and metabolic homeostasis [34]. Isoleucine is also considered a nitrogen donor to produce α-keto-β-methylvalerate, α-keto acid, and glutamate, which is a precursor for the inhibitory neurotransmitter γ-aminobutyric acid (GABA). Catabolism of isoleucine ultimately produces acetyl CoA and succinyl CoA, which can both be fed into the Krebs cycle for energy production [34,35]. Although isoleucine-containing dipeptides were differentially utilised between the WT and Ts1Cje neurospheres, the difference in utilisation of L-isoleucine itself was not found to be significant. This suggests that the dysregulated metabolism of isoleucine-containing dipeptides in Ts1Cje neurospheres is possibly attributed to defects in the enzymes involved in dipeptide hydrolysis or dipeptide transporters that internalise the dipeptides.

Clinically, reduced level of leucine was documented in the plasma of adults with DS as compared to healthy subjects, although the direct implication of this remains unclear [33]. The present study also reveals that the leucine-containing dipeptides were metabolised lower in the Ts1Cje neurospheres compared to the WT neurospheres. Leucine is a BCAA that was previously suggested to serve an important function as an amino group donor for the synthesis of glutamate [36]. The role of leucine in Ts1Cje, however, has not been explored and how leucine-containing dipeptides are utilised in Ts1Cje neurospheres is unknown. Because L-leucine itself was not found to be a significant differentially metabolised substrate in the current study, it can, therefore, be hypothesised that the leucine-containing dipeptides are also degraded to produce intermediates for protein synthesis and downstream energy metabolism pathways [37]. For example, leucine metabolism can ultimately produce acetyl CoA, which can be fed into the Krebs cycle.

In the present study, four tryptophan-containing dipeptides (Glu-Trp, Pro-Trp, Trp-Trp and Trp-Leu) were also found to be significantly differentially utilised by Ts1Cje-derived NSPCs. Tryptophan is an essential amino acid, as it cannot be synthesised in the body and is therefore obtained through diet. It is predominantly used for protein synthesis and as a precursor for kynurenine (KYN) and serotonin metabolic pathways [38]. Dysregulated tryptophan metabolism has been implicated in various neurological disorders, including Alzheimer’s disease, autism and depression [39–41]. In the KYN pathway, tryptophan is first oxidised either by the enzyme tryptophan 2,3-dioxygenase (TDO2), indoleamine 2,3-dioxygenase 1 (IDO-1) or IDO-2. This metabolic pathway ultimately produces the co-factor nicotinamide adenine dinucleotides (NAD^+^) and neuroactive intermediates such as quinolinic acid, the NMDA receptor agonist, kynurenic acid, the NMDA receptor antagonist and picolinic acid, a neuroprotectant [42].

Powers and colleagues [32] reported abnormal levels of KYN pathway intermediates, particularly KYN and the neurotoxic quinolinic acid in plasma of DS patients compared to control patients. This was the result of overactive IFN-γ signalling, leading to an increased expression of IDO1 and elevated levels of KYN [32]. Tryptophan catabolism through the serotonin pathway, where the initial step is catalyzed by tryptophan hydroxylase, results in the production of NADH. On the other hand, tryptophan catabolism along the KYN pathway leads to the production of NAD^+^, which is a precursor of NADH [43]. This suggests that altered tryptophan metabolism can be the result of dysregulations in either one of the pathways. However, the results in the present study revealed that utilisation of L-tryptophan was not significantly different between WT and Ts1Cje neurospheres. Therefore, it is also possible that utilisation of L-tryptophan as a single amino acid itself was not affected in the embryonic Ts1Cje neurospheres, but rather the dipeptide hydrolysis or cellular uptake of some dipeptides containing tryptophan that was altered.

While a couple of substrates were significantly poorly utilised by Ts1Cje neurospheres, some dipeptides (Asp-Glu, Glu-Tyr, Gly-Asp, Pro-Hyp), containing aspartic acid (Asp), proline (Pro), hydroxyproline (Hyp), alanine (Ala) and glutamate (Glu) were significantly more utilised in the Ts1Cje neurospheres instead. Although the specific function of each of these amino acids in neural progenitor cells of Ts1Cje mice are not known, it is postulated that the dipeptides are degraded to release the individual amino acids for utilisation in downstream metabolic pathways. Glutamate, on the other hand, is a known excitatory neurotransmitter in the brain that has been suggested to regulate the proliferation of neural precursor cells in developing rodent and human brains [44–46]. The level of glutamate was reported to be significantly lower in the adult hippocampus of an alternative DS mouse model, Ts2 mice, as well as the skeletal muscle isolated from adult Ts1Cje mice [19,47]. The disparity between findings from the previous studies and that from the present study could be due to utilisation of samples from adult mice as opposed to the embryonic sample used in this study. Regardless, these findings together indicate the perturbation of glutamate metabolism in Ts1Cje mice. Metabolism of amino acids and dipeptides has not been fully characterised in the Ts1Cje mouse brain, therefore, their functions are still poorly understood. Further characterisation and investigation of amino acids in Ts1Cje mice are required to understand their role in impaired proliferation and differentiation of NSPCs in this DS mouse model. In future, Biolog PM experiments could be extended to neurospheres derived from other Down syndrome mouse models such as Ts65Dn, Ts1Rhr to reveal a higher degree of representation of the true metabolic activity detected in the Ts1Cje neurospheres.

The Biolog PM analysis also revealed a lower utilisation of G6P in the Ts1Cje embryonic neurospheres compared to the WT neurospheres, suggesting that altered G6P metabolic pathways could contribute to defects in Ts1Cje embryonic NSPCs. G6P is involved in glycolysis and pentose phosphate pathway, which have been known to play a role in the survival and maintenance of NSPCs. There is limited knowledge on alterations in energy metabolic pathways, specifically in G6P metabolism in NSPCs of embryonic Ts1Cje neurospheres. Therefore, neurosphere supplementation assay was used to explore the role of G6P metabolic pathways in governing Ts1Cje NSPCs. 6PGA, an intermediate of the pentose phosphate pathway, was used as supplementation for neurosphere culture. The diameters of the neurospheres were then measured to assess the growth capacity at different concentrations of supplementation. Without 6PGA supplementation, the size of Ts1Cje neurospheres is significantly smaller than that of WT neurospheres, suggesting that the lower neurosphere-generating potential in Ts1Cje could be associated with a reduction in NSPC proliferation. Upon increasing concentration of 6PGA supplementation, the mean diameter of Ts1Cje is getting similar to that of WT neurospheres suggesting the ability of 6PGA in improving the proliferative capacity of NSPCs proliferation and neurosphere-generating potential through the energy-producing PPP.

Altered PPP may potentially contribute to the defective proliferation of embryonic Ts1Cje NSPCs and neurosphere generation, but the mechanistic contribution of PPP requires further investigation. The reliance of embryonic NSPCs on PPP is also supported by a previous study through inhibition of PPP with 6-aminonicotinamide (6-AN), which prevents G6P from entering PPP, leading to an approximately 60% decrease in survival of embryonic NSPCs [48]. The oxidative arm of PPP predominantly generates reduced NADPH, which functions to maintain reduced levels of glutathione (GSH) and reduce cellular oxidative stress. Intermediates from the non-oxidative arm of PPP, including ribose-5-phosphate and xylulose-5-phosphate, serve as the molecular backbone for nucleic acid and nucleotide synthesis [49,50]. Since PPP helps to control oxidative stress, findings from this current study could presumably suggest that disrupted PPP leads to accumulation of ROS in Ts1Cje neurospheres and hence increase cellular damage and death. Accumulating evidence has shown that increased oxidative stress contributes to the cognitive phenotypes observed in DS, and that it may occur at an early stage [51,52]. Previously, an elevated level of reactive oxygen species (ROS) was also observed in neurons and astrocytes cultured from embryonic Ts1Cje mice [53]. Increased level of lipid peroxidation, a marker for oxidative stress, was also found in the brains of Ts1Cje mice and was associated with accumulation of copper [54,55]. Together, these findings highlight the involvement of oxidative stress in the cognitive abnormalities in Ts1Cje mice.

Findings from this present study support previous results that indicate the contribution of dysregulated metabolic pathways in impaired proliferation and differentiation of Ts1Cje neurospheres. Functional enrichment analysis of probes that were overexpressed in adult Ts1Cje neurospheres revealed genes involved in cellular energy production and metabolism, suggesting a role for dysregulated metabolic pathways in Ts1Cje cells [12]. In a later study, functional analysis on downregulated genes in E15.5 embryonic Ts1Cje brains identified enrichment of genes associated with solute carrier (SLC)-amino acid transmembrane transporters [56]. This finding supports the altered amino acid utilisation by embryonic Ts1Cje neurospheres reported in the present study. A proteomic profiling study also revealed increased protein expression of pyruvate kinase (PK) in embryonic Ts1Cje brain compared to WT littermates ^57^ PK is an enzyme involved in glycolysis, which converts phosphoenopyruvate to pyruvate, yielding one molecule of ATP. Altogether, previous findings provide transcriptomic and proteomic evidence to support the involvement of disrupted energy metabolism observed in this present study. However, whether the abberations in metabolic pathways is a direct effect of the dysregulated genes in the trisomic region of Ts1Cje mice is still unclear and therefore warrants further investigation.

There has been studies reported on the genetically inspired biological distinctions between male and female mouse model, including Down syndrome mice such as Trisomic Dp(10)1Yey [57] and Ts65Dn [58,59]. In the current study, the metabolic phenotypic microarray profiling was performed on the Ts1Cje embryos without gender-specific, hence, for future study, the gender gap should be taken into consideration.

In summary, the present study has demonstrated differences in substrate utilisation pattern between the embryonic Ts1Cje and WT neurospheres through the use of Biolog PM technology. A prominent finding from this global metabolic profiling is the decreased utilisation of G6P, which is part of the PPP. The potential involvement of altered PPP in Ts1Cje NSPCs is subsequently confirmed by the ability of 6PGA to rescue neurosphere-generating potential in the embryonic Ts1Cje neurospheres. These results introduce the potential role of disrupted energy metabolism in the deficits observed in embryonic NSPCs derived from Ts1Cje mice. Furthermore, metabolic profiling also highlights alternative sources that can be utilised by embryonic NSPCs for energy production. This present study only provides a general picture of the metabolic activity of Ts1Cje neurospheres and insights on underlying dysregulations in neurogenesis during early brain development in DS, which to a certain extent, complement previous genomic, transcriptomic and proteomic characterisation of Ts1Cje neurospheres. To better understand energy metabolism in Ts1Cje neurospheres, further validation of the significant differentially utilised substrates is required. A more comprehensive investigation using the metabolomics approach could enhance the understanding of the causes of defects in proliferation and differentiation of embryonic NSPCs derived from Ts1Cje mice. This could facilitate future research on nutritional intervention for the management of neurocognitive phenotypes observed in individuals with DS.

## Supporting information

S1 File (XLSX)

S1 Raw data (XLSX)

S2 Raw data (XLSX)

S3 Raw data (XLSX)

S4 Raw data (XLSX)

## References

[1] Busciglio J, Capone G, O’Bryan J, O’Byran JP, Gardiner KJ. Down syndrome: genes, model systems, and progress towards pharmacotherapies and clinical trials for cognitive deficits. Cytogenet Genome Res. 2013; 141: 260–271. 10.1159/000354306 24008277

[2] Rachidi M, Lopes C. Molecular and cellular mechanisms elucidating neurocognitive basis of functional impairments associated with intellectual disability in Down syndrome. Am J Intellect Dev Disabil. 2010; 115: 83–112. 10.1352/1944-7558-115.2.83 20441388

[3] Sago H, Carlson EJ, Smith DJ, Kilbridge J, Rubin EM, Mobley WC, et al Ts1Cje, a partial trisomy 16 mouse model for Down syndrome, exhibits learning and behavioral abnormalities. Proc Natl Acad Sci U S A. 1998; 95: 6256–6261. 10.1073/pnas.95.11.6256 9600952 PMC27649

[4] Bala U, Leong MP-Y, Lim CL, Shahar HK, Othman F, Lai M-I, et al Defects in nerve conduction velocity and different muscle fibre-type specificity contribute to muscle weakness in Ts1Cje Down syndrome mouse model. PLoS One. 2018; 13: e0197711 10.1371/journal.pone.0197711 29795634 PMC5967806

[5] Olson LE, Roper RJ, Baxter LL, Carlson EJ, Epstein CJ, Reeves RH. Down syndrome mouse models Ts65Dn, Ts1Cje, and Ms1Cje/Ts65Dn exhibit variable severity of cerebellar phenotypes. Dev Dyn. 2004; 230: 581–589. 10.1002/dvdy.20079 15188443

[6] Laffaire J, Rivals I, Dauphinot L, Pasteau F, Wehrle R, Larrat B, et al Gene expression signature of cerebellar hypoplasia in a mouse model of Down syndrome during postnatal development. BMC Genomics. 2009; 10: 138 10.1186/1471-2164-10-138 19331679 PMC2678156

[7] Noctor SC, Martinez-Cerdeño V, Kriegstein AR. Neural stem and progenitor cells in cortical development. Novartis Found Symp. 2007; 288: 59–73. 18494252

[8] Urbán N, Guillemot F. Neurogenesis in the embryonic and adult brain: same regulators, different roles. Front Cell Neurosci. 2014; 8: 396 10.3389/fncel.2014.00396 25505873 PMC4245909

[9] Stagni F, Giacomini A, Emili M, Guidi S, Bartesaghi R. Neurogenesis impairment: An early developmental defect in Down syndrome. Free Radic Biol Med. 2018; 114: 15–32. 10.1016/j.freeradbiomed.2017.07.026 28756311

[10] Bhattacharyya A, McMillan E, Chen SI, Wallace K, Svendsen CN. A critical period in cortical interneuron neurogenesis in down syndrome revealed by human neural progenitor cells. Dev Neurosci. 2009; 31: 497–510. 10.1159/000236899 19738365 PMC2818457

[11] Reynolds BA, Weiss S. Generation of neurons and astrocytes from isolated cells of the adult mammalian central nervous system. Science (80-). 1992; 255: 1707–1710. 10.1126/science.1553558 1553558

[12] Hewitt CA, Ling KH, Merson TD, Simpson KM, Ritchie ME, King SL, et al Gene network disruptions and neurogenesis defects in the adult Ts1Cje mouse model of down syndrome. Aziz SA, editor. PLoS One. 2010; 5: e11561 10.1371/journal.pone.0011561 20661276 PMC2905390

[13] Lee HC, Tan KL, Cheah PS, Ling KH. Potential Role of JAK-STAT Signaling Pathway in the Neurogenic-to-Gliogenic Shift in Down Syndrome Brain. Neural Plast. 2016; 2016. 10.1155/2016/7434191 PMC4737457 26881131

[14] Lee H-C, Yusof HH Md, Leong MP-Y, Zainal Abidin S, Seth EA, Hewitt CA, et al Gene and protein expression profiles of JAK-STAT signalling pathway in the developing brain of the Ts1Cje down syndrome mouse model. Int J Neurosci. 2019; 129: 871–881. 10.1080/00207454.2019.1580280 30775947

[15] Hsia DY-Y, Justice P, Smith GF, Dowben RM. Down’s Syndrome. Am J Dis Child. 1971; 121: 153 5542854

[16] Labudova O, Kitzmueller E, Rink H, Cairns N, Lubec G. Increased phosphoglycerate kinase in the brains of patients with Down’s syndrome but not with Alzheimer’s disease. Clin Sci (Lond). 1999; 96: 279–285. 10029564

[17] Simo M, Garcia JR, Hernadez I, Escanilla A, Boada M, Lomena F. Evaluation of cerebral glucose metabolism with Positron Emission Tomography in subjects with Down syndrome. Int Med J Down Syndr. 2004; 8: 23–28.

[18] Smigielska-Kuzia J, Sobaniec W. Brain metabolic profile obtained by proton magnetic resonance spectroscopy HMRS in children with Down syndrome. Adv Med Sci. 2007; 52 Suppl 1: 183–187. 18229661

[19] Lim CL, Bala U, Leong MP-Y, Yap IKS, Stanslas J, Ramasamy R, et al Perturbed metabolic profiles associated with muscle weakness seen in adult Ts1Cje mouse model of Down syndrome. Jpn J Vet Res. 2019; 67: 111–118.

[20] Bochner BR, Siri M, Huang RH, Noble S, Lei X-H, Clemons PA, et al Assay of the multiple energy-producing pathways of mammalian cells. PLoS One. 2011; 6: e18147 10.1371/journal.pone.0018147 21455318 PMC3063803

[21] Shea A, Wolcott M, Daefler S, Rozak D. Biolog phenotype microarrays. Methods Mol Biol. 2012; 881: 331–373. 10.1007/978-1-61779-827-6_12 22639219

[22] Ling K-H, Hewitt CA, Tan K-L, Cheah P-S, Vidyadaran S, Lai M-I, et al Functional transcriptome analysis of the postnatal brain of the Ts1Cje mouse model for Down syndrome reveals global disruption of interferon-related molecular networks. BMC Genomics. 2014; 15: 624 10.1186/1471-2164-15-624 25052193 PMC4124147

[23] Vehkala M, Shubin M, Connor TR, Thomson NR, Corander J. Novel R pipeline for analyzing biolog phenotypic microarray data. PLoS One. 2015; 10: e0118392 10.1371/journal.pone.0118392 25786143 PMC4365023

[24] Obel LF, Müller MS, Walls AB, Sickmann HM, Bak LK, Waagepetersen HS, et al Brain glycogen-new perspectives on its metabolic function and regulation at the subcellular level. Front Neuroenergetics. 2012; 4: 3 10.3389/fnene.2012.00003 22403540 PMC3291878

[25] Brunet JF, Allaman I, Magistretti PJ, Pellerin L. Glycogen metabolism as a marker of astrocyte differentiation. J Cereb Blood Flow Metab. 2010; 30: 51–55. 10.1038/jcbfm.2009.207 19809466 PMC2949090

[26] Mergenthaler P, Lindauer U, Dienel GA, Meisel A. Sugar for the brain: The role of glucose in physiological and pathological brain function. Vol. 36, Trends in Neurosciences. 2013 p. 587–597. 10.1016/j.tins.2013.07.001 23968694 PMC3900881

[27] Horie N, Moriya T, Mitome M, Kitagawa N, Nagata I, Shinohara K. Lowered glucose suppressed the proliferation and increased the differentiation of murine neural stem cells in vitro. FEBS Lett. 2004; 571: 237–242. 10.1016/j.febslet.2004.06.085 15280049

[28] de Koning TJ, Snell K, Duran M, Berger R, Poll-The B-T, Surtees R. L-serine in disease and development. Biochem J. 2003; 371: 653–661. 10.1042/BJ20021785 12534373 PMC1223326

[29] Jiang R, Ren T-J, Qiang R, Wang G-H, Sun L, Zhao G-W, et al L-Serine Treatment May Improve Neurorestoration of Rats after Permanent Focal Cerebral Ischemia Potentially Through Improvement of Neurorepair. PLoS One. 2014; 9: e93405 10.1371/journal.pone.0093405 24671106 PMC3966884

[30] Stříšovský K, Jirásková J, Bařinka C, Majer P, Rojas C, Slusher BS, et al Mouse brain serine racemase catalyzes specific elimination of L-serine to pyruvate. FEBS Lett. 2003; 535: 44–48. 10.1016/s0014-5793(02)03855-3 12560076

[31] Scolari MJ, Acosta GB. D-serine: A new word in the glutamatergic neuro-glial language. Amino Acids. 2007; 33: 563–574. 10.1007/s00726-006-0481-0 17245616

[32] Powers RK, Culp-Hill R, Ludwig MP, Smith KP, Waugh KA, Minter R, et al Trisomy 21 activates the kynurenine pathway via increased dosage of interferon receptors. Nat Commun. 2019; 10: 1–11. 10.1038/s41467-018-07882-8 31628327 PMC6800452

[33] Coppus AW, Fekkes D, Verhoeven WMA, Tuinier S, Egger JIM, Van Duijn CM. Plasma amino acids and neopterin in healthy persons with Down’s syndrome. J Neural Transm. 2007; 114: 1041–1045. 10.1007/s00702-007-0656-1 17401539 PMC2794348

[34] Sperringer JE, Addington A, Hutson SM. Branched-Chain Amino Acids and Brain Metabolism. Neurochem Res. 2017; 42: 1697–1709. 10.1007/s11064-017-2261-5 28417264

[35] Manoli I, Venditti CP. Disorders of branched chain amino acid metabolism. Transl Sci Rare Dis. 2016; 1: 91–110. 10.3233/TRD-160009 29152456 PMC5685199

[36] Yudkoff M, Daikhin Y, Nissim I, Horyn O, Luhovyy B, Lazarow A, et al Brain Amino Acid Requirements and Toxicity: The Example of Leucine. J Nutr. 2005; 135: 1531S–1538S. 10.1093/jn/135.6.1531S 15930465

[37] Kurbat MN, Lelevich V V. Metabolism of amino acids in the brain. Neurochem J. 2009; 3: 23–28.

[38] Richard DM, Dawes MA, Mathias CW, Acheson A, Hill-Kapturczak N, Dougherty DM. L-Tryptophan: Basic Metabolic Functions, Behavioral Research and Therapeutic Indications. Int J Tryptophan Res. 2009; 2: 45–60. 10.4137/ijtr.s2129 20651948 PMC2908021

[39] Bryn V, Verkerk R, Skjeldal OH, Saugstad OD, Ormstad H. Kynurenine Pathway in Autism Spectrum Disorders in Children. Neuropsychobiology. 2017; 76: 82–88. 10.1159/000488157 29694960

[40] Gulaj E, Pawlak K, Bien B, Pawlak D. Kynurenine and its metabolites in Alzheimer’s disease patients. Adv Med Sci. 2010; 55: 204–211. 10.2478/v10039-010-0023-6 20639188

[41] Oxenkrug GF. Tryptophan-kynurenine metabolism as a common mediator of genetic and environmental impacts in major depressive disorder: The serotonin hypothesis revisited 40 years later. Isr J Psychiatry Relat Sci. 2010; 47: 56–63. 20686200 PMC3021918

[42] Jones SP, Guillemin GJ, Brew BJ. The kynurenine pathway in stem cell biology. Int J Tryptophan Res. 2013; 6: 57–66. 10.4137/IJTR.S12626 24092986 PMC3782398

[43] Boccuto L, Chen C-F, Pittman AR, Skinner CD, McCartney HJ, Jones K, et al Decreased tryptophan metabolism in patients with autism spectrum disorders. Mol Autism. 2013; 4: 16 10.1186/2040-2392-4-16 23731516 PMC3680090

[44] Haydar TF, Wang F, Schwartz ML, Rakic P. Differential modulation of proliferation in the neocortical ventricular and subventricular zones. J Neurosci. 2000; 20: 5764–5774. 10.1523/JNEUROSCI.20-15-05764.2000 10908617 PMC3823557

[45] Luk KC, Kennedy TE, Sadikot AF. Glutamate promotes proliferation of striatal neuronal progenitors by an NMDA receptor-mediated mechanism. J Neurosci. 2003; 23: 2239–2250. 10.1523/JNEUROSCI.23-06-02239.2003 12657683 PMC6742023

[46] Suzuki M, Nelson AD, Eickstaedt JB, Wallace K, Wright LS, Svendsen CN. Glutamate enhances proliferation and neurogenesis in human neural progenitor cell cultures derived from the fetal cortex. Eur J Neurosci. 2006; 24: 645–653. 10.1111/j.1460-9568.2006.04957.x 16848797

[47] Kaur G, Sharma A, Xu W, Gerum S, Alldred MJ, Subbanna S, et al Glutamatergic transmission aberration: A major cause of behavioral deficits in a murine model of Down’s syndrome. J Neurosci. 2014; 34: 5099–5106. 10.1523/JNEUROSCI.5338-13.2014 24719089 PMC3983795

[48] Candelario KM, Shuttleworth CW, Cunningham LA. Neural stem/progenitor cells display a low requirement for oxidative metabolism independent of hypoxia inducible factor-1alpha expression. J Neurochem. 2013; 125: 420–429. 10.1111/jnc.12204 23410250 PMC4204647

[49] Kim DY, Rhee I, Paik J. Metabolic circuits in neural stem cells. Cell Mol Life Sci. 2014; 71: 4221–4241. 10.1007/s00018-014-1686-0 25037158 PMC4394599

[50] Stincone A, Prigione A, Cramer T, Wamelink MMC, Campbell K, Cheung E, et al The return of metabolism: biochemistry and physiology of the pentose phosphate pathway. Biol Rev Camb Philos Soc. 2015; 90: 927–963. 10.1111/brv.12140 25243985 PMC4470864

[51] Perluigi M, di Domenico F, Fiorini A, Cocciolo A, Giorgi A, Foppoli C, et al Oxidative stress occurs early in Down syndrome pregnancy: A redox proteomics analysis of amniotic fluid. Proteomics Clin Appl. 2011; 5: 167–178. 10.1002/prca.201000121 21360684

[52] Cenini G, Dowling ALS, Beckett TL, Barone E, Mancuso C, Murphy MP, et al Association between frontal cortex oxidative damage and beta-amyloid as a function of age in Down syndrome. Biochim Biophys Acta. 2012; 1822: 130–138. 10.1016/j.bbadis.2011.10.001 22009041 PMC3260028

[53] Shukkur EA, Shimohata A, Akagi T, Yu W, Yamaguchi M, Murayama M, et al Mitochondrial dysfunction and tau hyperphosphorylation in Ts1Cje, a mouse model for Down syndrome. Hum Mol Genet. 2006; 15: 2752–2762. 10.1093/hmg/ddl211 16891409

[54] Ishihara K, Amano K, Takaki E, Ebrahim AS, Shimohata A, Shibazaki N, et al Increased lipid peroxidation in Down’s syndrome mouse models. J Neurochem. 2009; 110: 1965–1976. 10.1111/j.1471-4159.2009.06294.x 19645748

[55] Ishihara K, Yasui H, Nagasawa K, Kawashita E, Yamakawa K, Shimizu R, et al Copper accumulation in the brain causes the elevation of oxidative stress and less anxious behavior in Ts1Cje mice, a model of Down syndrome. Free Radic Biol Med. 2019; 134: 248–259. 10.1016/j.freeradbiomed.2019.01.015 30660502

[56] Guedj F, Pennings JLA, Ferres MA, Graham LC, Wick HC, Miczek KA, et al The fetal brain transcriptome and neonatal behavioral phenotype in the Ts1Cje mouse model of Down syndrome. Am J Med Genet A. 2015; 167: 1993–2008. 10.1002/ajmg.a.37156 PMC4844354 25975229

[57] Block A, Mahiuddin Ahmed M, Ranjitha Dhanasekaran A, Tong S, Gardiner KJ. Sex differences in protein expression in the mouse brain and their perturbations in a model of Down syndrome. Biol Sex Differ. 2015; 6: 1–18. 10.1186/s13293-014-0019-1 26557979 PMC4640233

[58] Martínez-Cué C, Noemi R, García E, Flórez J. Anxiety and panic responses to a predator in male and female Ts65Dn mice, a model for Down syndrome. Genes Brain Behav. 2006; 5: 413–422. 10.1111/j.1601-183X.2005.00175.x 16879635

[59] Martínez-Cué C, Baamonde C, Lumbreras M, Paz J, Davisson MT, Schmidt C, et al Differential effects of environmental enrichment on behavior and learning of male and female Ts65Dn mice, a model for Down syndrome. Behav Brain Res. 2002; 134: 182–200. 10.1016/s0166-4328(02)00026-8 12191805

